# Predictors of Mental Health after the First Wave of the COVID-19 Pandemic in Poland

**DOI:** 10.3390/brainsci11050544

**Published:** 2021-04-27

**Authors:** Piotr Długosz

**Affiliations:** Faculty of Social Sciences, Pedagogical University of Krakow, 30-084 Krakow, Poland; piotr.dlugosz@up.krakow.pl

**Keywords:** COVID-19, fear of COVID-19, mental health, emotional distress, social effects

## Abstract

The aim of the article is to determine the predictors of mental health among Polish society. Research was conducted after the first wave of the pandemic. Due to such an approach, it was possible to determine whether the secondary effects of the pandemic have impacted on mental health, in addition to socio-demographic and psychological factors. In order to gather the research material, the CAWI on-line survey method was applied and carried out within the framework of the Ariadna Research Panel on a sample of 1079 Poles, aged 15 and over. The FCV-19S scale, which is used to measure the fear of COVID-19 was applied in the measurement. It is a verified diagnostic instrument used to measure mental health across a range of countries. The results of a hierarchical regression analysis have shown that the factors which increase the level of fear of COVID-19 are demographic, social and psychological features, as well as attitudes towards the pandemic. The results of research indicate the significance of social context in the analysis, and contribute to the explanation of the effects of disasters and cataclysms.

## 1. Introduction

The COVID-19 pandemic, which suddenly occurred in Wuhan, China, in December 2019, has spread rapidly all over the world, resulting in a threat to life and health. On the basis of numerous meta-analyses, it is possible to determine a negative impact of the COVID-19 pandemic on the mental health of numerous societies [1,2,3,4,5].

Research conducted in numerous countries have revealed a deterioration of mental health and an increase in anxiety disorders [6,7,8,9]. By applying the FCV-19S fear of COVID-19 scale in the study, the relationship between the fear and demographic variables, such as age, gender, general health, marital status and belonging in a high-risk group has been revealed [9,10,11,12,13]. There is also evidence that health anxiety, regular media use and social media use have an impact on the fear of COVID-19 [14].

To date, the studies which indicate the relationship between the fear of COVID-19 with socio-demographic features, and studies which demonstrate how the fear of COVID-19 may negatively influence mental health and related disorders in the form of anxiety, stress and depression, are prevalent [15,16,17].

The fear of COVID-19 may be increased by the secondary effects of the pandemic. Unemployment, isolation and quarantine as well as a dramatic change in people’s lifestyle occurred together with the imposition of lockdown. The new situation has become a new source of fear, as people do not know whether they will be still employed, or when the pandemic will end. Therefore, it is worth supplementing the current studies on the impact of the COVID-19 pandemic with the knowledge on the risk factors increasing the fear of COVID-19. Demonstrating the social context in which individuals experience the fear of coronavirus and indicating the possible differences between various groups will be helpful in terms of determining the level of the threat to mental health, which has been given rise to by the pandemic. Gaps in knowledge and the need to fill them within the above-mentioned factors have been also indicated by the results of meta-analyses [18].

The fear also has an adaptive function, and acts as a signal for the body that a threat, which should be avoided, is approaching. It may reinforce the tendency to comply with safety norms, such as keeping social distance, washing hands and wearing masks [19]. Nonetheless, if the fear is too intense and lasts for too long, it may result in disorders such as phobias, post-traumatic stress disorder or even coronaphobia, and thus hinder social functioning [20].

Due to the fact that the new coronavirus is a serious threat, giving rise to fear reactions on a mass scale, a previously prepared instrument was used in its measurement, since the FCV-19S has good psychometric properties, and can be utilized in studies assessing the effects of the pandemic on the population’s mental health [6,13].

The assessment of the threat to the mental health of Poles was conducted after the first wave of the pandemic. Due to such an approach, it will be possible to verify whether the changes and hindrances to one’s daily life have had an impact on one’s mental health. It is a significant issue, as the findings to date indicate ambiguity. There are the results of a meta-analysis of longitudinal studies indicating that the quarantine and lockdown had a limited impact on the deterioration of the mental health of inhabitants of numerous countries [21]. There are also findings indicating that fear and depression were higher among adults who lost their income and jobs. Experiencing an income shock has been observed in such situations [22].

The article aims to verify whether secondary effects of the pandemic which arise from lockdown have impacted on the mental health of Poles, aside from socio-demographic features. The hypothesis that restrictions and nuisances introduced in order to stop the transmission of coronavirus will have a negative impact on mental health shall be proposed.

## 2. Methods

### 2.1. Participants and Procedure

The study was conducted on a representative nationwide Polish sample (*n* = 1079; 554 women; age range = 15 to 94, M = 42.4, SD = 16.7). The participants were recruited using the Ariadna Polish on-line panel (CAWI), which has over 110,000 active panel members, aged 15 and over. Participants were rewarded for their participation with points collected via their panel membership, the points being exchangeable for rewards in a pool of several hundred products offered by the panel organizers. All participants provided their informed consent and the study was conducted anonymously.

The selection of the research sample was carried out in two stages. In the first stage, the population was segregated into subgroups based on mutual exclusivity. Then, from such separated groups, respondents were selected based on the stratification model, including gender, age, place of residence and region. In the second stage, the respondents were recruited based on demographic data from the Central Statistical Office in Poland. All procedures were conducted in accordance with the ethical standards of the institutional and/or Polish national research committees, and with the 1964 Helsinki declaration and its later amendments or comparable ethical standards.

The studies were carried out between the 26th and the 30th of October 2020. On the day of the commencement of the research, there were 1584 cases of COVID-19 in Poland, whereas on the 30th of September there were 1552 cases. The research was conducted a day before the second wave of the pandemic, as from October the number of incidences increased dramatically and by the end of the month there were over 22,000 cases. Therefore, the research was conducted in the circumstances of a relative stability in the number of incidences after the summer break. It may be assumed that it was a perfect moment to capture the consequences of the pandemic after the first wave.

### 2.2. Demographic Characteristics

The research form included questions regarding the following demographic factors: gender, age, education, socio-economic status, marital status (Table 1).

In the research, the variables depicting the social context of the pandemic were used as well. Their characteristics and the obtained results are presented below.

#### 2.2.1. Social Capital

The measurement of social capital was conducted with the use of a modified instrument put forward by Putnam [23]. The social capital variable consists of 5 items. The respondents were asked if they can count on help from their families and friends in case of (1) looking for a job; (2) being in a difficult financial situation; (3) the need to be taken care of; (4) dealing with official matters; (5) the need to explain a complicated matter. Scores range from 0 to 5. The average social capital variable was 3.4, (SD = 1.6).

#### 2.2.2. Loss of Economic Resources

Four questions, demanding yes/no answers, were included in the research. The questions were as follows-If, during the pandemic, the researched individual had experienced the loss of job, a decrease in the number of working hours, taken up remote work, or focused on looking after their children at home. The changes on the labour market were most often based on taking up remote work (15%), a decrease in one’s working hours (14%), the loss of job (6%) and focusing on looking after children (6%). Scores ranged from 0 to 4. The higher the score, the bigger the loss of economic resources. The average on the variable was 0.4, SD = 0.5.

#### 2.2.3. Fear of Losing Job

The fear of losing job was measured with the use of an ordinal scale, by asking the respondents whether they reckon that they may lose their current jobs due to lockdown. The following answers have been obtained: definitely yes (5%), rather yes (16%), rather not (35%), definitely not (24%), hard to say (21%).

#### 2.2.4. Life Changes

Changes in the lifestyle variable of participating individuals that may have been impacted by the pandemic were assessed by the following four questions: (1) cancellation of vacation (45%), (2) resignation from social and family meetings (39%), (3) resignation from participation in cultural events (56%), (4) resignation from participation in religious services (32%). Scores ranged from 0 to 4. The higher the score, the higher the level of life changes. The average value of this variable was 1.7, SD = 1.2.

#### 2.2.5. Need Deprivation

The deprivation of needs variable has been prepared on the basis of answers pertaining to the impact of the COVID-19 pandemic on various aspects of social functioning. The instrument measuring the deprivation of needs consists of 8 questions. The distribution of answers indicating the level of deprivation is presented below. The pandemic had a negative impact on satisfying the following needs: (1) food (10%), (2) spending free time with one’s family (19%), 3) performing occupational duties (17%), (4) financial conditions (26%), (5) culture (40%), (6) education (27%), (7) leisure (35%), (8) health (37%). The score on the index ranges from 0 to 8. The average on the variable was 2.1, SD = 2.1. The higher the score, the higher the level of deprivation.

#### 2.2.6. Mindset Change

The pandemic, apart from a decrease in the sense of social security, has led to the loss of control over one’s life and events occurring in it. A Mindset Change Index has been made by summing up the answers indicating the loss of: (1) the sense of stability and certainty (37%), the sense that I will achieve my goals (28%), the sense of certainty in one’s workplace (24%), the sense of control over life events (31%), the sense of being calm and carefree (40%), the sense of security in a relationship or marriage (12%). Scores ranged from 0 to 6. The higher the score, the bigger the loss of psychological resources. The average on the variable was 1.7, SD = 1.6.

#### 2.2.7. Life Position Decrease

In order to verify the extent to which the coronavirus epidemic undermined the life position of the respondents, the Cantril Scale (CS) [24] was used to ask about the experienced life position on a scale ranging from 1 to 10 (where 1 is the worst possible life and 10 is the best possible life). The respondents were asked to compare the position they had before the pandemic and the position they have during quarantine. The average result of the life position before COVID-19 was 6.92, SD = 1.8, whereas during the quarantine the result was 6.47, SD = 1.9. The indication of a decrease in the quality of life was calculated on the basis of the difference between the position estimated on the day of the research, and the position before the pandemic. Scores on this index range from −9 to 9. Higher results mean a bigger decrease in one’s life position. The average on the scale was 0.4, SD = 1.6.

#### 2.2.8. Attitudes towards the COVID-19 Pandemic

By means of an open-ended question, the respondents were asked to estimate the probability of becoming infected with coronavirus on the scale from 0 to 100. The average chance of becoming infected has been estimated to be about 40.9%, SD = 25.8.

The scale used to measure the level of interest in the pandemic consists of 4 scores. The score ranges from 0 to 4. The responses range from “I’m not interested at all” to “I’m very interested”. Higher values indicate a higher interest. The average on the scale was 2.9, SD = 0.7.

#### 2.2.9. Psychological Variables

##### Neuroticism

The scale used to measure neuroticism was constructed according to the model of the Neuroticism Scale of the Eysenck Personality Questionnaire-Revised (EPQ-R) [25]. It consists of 13 items. The response to each item was recorded according to a 5-point Likert scale ranging from 1 (definitely not) to 5 (definitely yes). Scores on this scale ranged from 13 to 65, with higher scores indicating higher levels of neuroticism. The coefficient alpha in the present study was 0.87. The average (EPQ-R) was 38.0, SD = 8.

##### Strategies for Coping with Stress

The construct put forward by Lazarus and Folkman [26] was used to measure the strategies for coping with stress. The problem-focused strategy is aimed at finding an actual solution to the problem, changing the situation for the better. The emotion-focused strategy is aimed at a change in the manner of experiencing a stressful situation emotionally. In order to measure the strategies, the scale used in longitudinal studies in Poland was applied [27].

##### Stress

In order to measure the dependent variable, the Kessler Psychological Distress Scale (K10) which measures the symptoms of anxiety and depression in the society was used [28]. The scale consists of 10 items and its Cronbach α = 0.948. The response for each item was recorded according to a 5-point Likert scale ranging from 1 (All of the time) to 5 (None of the time). The minimum value on the scale was 10, whereas the maximum value was 50. The average value for the researched sample was M = 22.9, SD = 8.1.

##### Psychological Well-Being

In order to measure well-being, an ordinal scale consisting of 5 items was used. Its value ranged from very dissatisfied (1) to very satisfied (5). The average value for the researched sample was M = 3.71, SD = 1.03.

##### Fear of COVID-19

The analysed dependent variable was mental health, measured with the Fear of COVID-19 Scale (FCV-19S) developed by [6,12]. The items of the FCV-19S were constructed based on extensive reviews of existing scales on fears, expert evaluations and participant interviews [6]. The FCV-19S consists of 10 items (Table 2).

The response for each item was recorded according to a 5-point Likert scale ranging from 1 (definitely not) to 5 (definitely yes). The overall score for fear (ranging from 10 to 50) was obtained by adding up each item score. The higher the overall score, the greater the fear of COVID-19. Cronbach α = 0.93. The average fear on COVID-19 Scale was 23.7, SD = 9.

The results presented in the table indicate that Polish society displays mainly cognitive-subjective aspects of the fear of COVID-19 [29]. The greatest anxiety among the respondents was caused by the unpredictability of coronavirus, distress, and worrying (addressed in items 5, 2 and 3). The respondents also experienced reactions of a psychosomatic origin to a lesser extent (insomnia, sweaty hands, addressed in items 9 and 6). The results obtained from the Polish research sample are similar to those obtained in other studies [9,11,13].

## 3. Results

The research into the mental health predictors of Polish nationals after the first wave of COVID-19 took place in two stages. In the first stage of analysis, a correlation analysis was carried out in order to observe the relationship between variables. In the second stage, a hierarchical regression analysis was conducted in four steps, which allowed for a detailed identification of mental health risk factors.

The correlation analysis shown in Table 3 indicates that the fear of COVID-19 is correlated with age, financial status, fear of the loss of employment, mindset change index, and life position decrease index. Stress, the level of interest in media, and the fear of becoming infected have a stronger impact. Neuroticism and strategies for coping with the threat of COVID-19 also have a significant impact on the level of fear of COVID-19. Therefore, the obtained results of the correlation analysis confirm the fact that social context has a significant impact on the mental health of individuals during the COVID-19 pandemic.

The hierarchical regression analysis was used to find the predictors of mental health (Table 4). In the first stage, socio-demographic variables were introduced. In the second stage, the variables measuring the negative effects of lockdown were introduced, in the third stage, the attitudes towards COVID-19 were introduced, and, finally, in the fourth stage, psychological variables were introduced.

The results of the hierarchical regression analysis indicate that demographic variables explain 3% of the fear of COVID-19 variance. Higher levels of stress were experienced by older people, and those who evaluate their financial standing as worse.

Introducing variables measuring the social effects of lockdown to the model resulted in a significant change in corrected R2, (delta corrected R2 = 0.115; F change (6721) = 22.999, *p* < 0.000), and indicates that introducing those variables has increased the level of explained variance to 18%. The levels of fear of COVID-19 increased in tandem with age; it was negatively correlated with education, and positively correlated with the fear of unemployment, the experienced limitations in life activities, and deprivation of needs. In the third stage, upon introducing attitudes towards COVID-19, the level of explained variance was 36%. The change in the explained variance was statistically relevant. A significant change in corrected R2, (delta corrected R2 = 0.187; F change (2719) = 107.769, *p* < 0.000) was observed. In this model, age and education have retained their influence. The evaluation of financial status was also statistically relevant. The impact of variables measuring the effects of lockdown: the fear of unemployment, limitations in possible activities, deprivation of needs, and a decrease in life position has remained as well. The introduced new variables, i.e., the level of interest in information on COVID-19 and the level of fear of becoming infected turned out to be significant predictors of the fear of COVID-19.

In the fourth stage, the introduced psychological variables increased the percentage of the explained variance to 48%. The change in the explained variance was statistically relevant. A significant change in corrected R2, (delta corrected R2 = 0.126; F change (5714) = 36.179, *p* < 0.000) was observed. In this model, the impact of all the statistically relevant variables from the previous stage of analysis has remained. Out of the subsequent variables introduced to the model, stress, problem-focused strategies and satisfaction with life turned out to be positively correlated with fear.

## 4. Discussion

The results of analyses confirm the hypothesis proposed in the introduction. The hypothesis states that the life-and-health-threatening COVID-19 has impacted on the mental health of Poles. Social context in which individuals function during the lockdown is significant as well. A dramatic change in lifestyle, a forced change in life habits, a decrease in the quality of life, inconvenient limitations introduced as a result of the quarantine, and deprivation of needs are all secondary effects of the pandemic. Correlation analysis and hierarchical regression analysis confirm the increase in the fear of COVID-19 as a result of the loss of resources during lockdown. This phenomenon is well explained by the Conservation of Resources Theory (COR) proposed by Hobfoll [30,31]. The pandemic has led to the loss of various resources, such as: health, a high quality of life, job, money and friends. Moreover, it prevented many individuals from satisfying their values, such as leisure, free time, travelling, entertainment, security and family. It has been observed that people with a lesser number of resources, that is, older people and poor people, have experienced a deterioration of their mental health.

The theory of life change also serves the purpose of explaining secondary effects the pandemic had on mental health [32]. It demonstrates how lots of people had to adapt to a considerably changed reality as a result of the imposed lockdown. The more changes in the daily lives of individuals occurred, the stronger was the fear experienced in relation to COVID-19.

The conducted studies have shown that the social context of the pandemic is significant and thus should be taken into account while attempting to estimate mental health risk factors [14,33]. The fact that the COVID-19 pandemic is a phenomenon which concerns almost everyone without exceptions is particularly crucial. It constitutes a collective trauma [34,35,36,37].

Before moving to an in-depth discussion of the results of the analyses, it is necessary to stress the fact that demographic factors had a small impact on the level of fear. The older the respondents, the higher the level of anxiety. It may be explained by the fact that COVID-19 poses a threat mainly to older people. The risk of death and dangerous complications increases together with age [38]. Therefore, older people experience higher levels of fear. The obtained results are convergent with the results of the study into the fear of COVID-19 [39]. It is worth adding that there are findings indicating higher levels of fear among young people [40]. There are also results of research from eleven countries which indicate lower levels of fear among people aged 18 and over, and 60 and over. The obtained results are explained by the fact that the youngest respondents do not experience a deep fear, since they will not lose their jobs and income. On the other hand, older respondents may think that they do not have much to lose, as they have lived for a long time and may be reonciled to the idea of death. Therefore, the fear of COVID-19 among the oldest respondents may be lower [41]. These issues require further studies.

Individuals with lower education and a generally lower social status experienced higher levels of stress, which may be explained by the fact that their sensitivity threshold for the loss of financial resources is stronger [31]. They are more anxious about the fact that lockdown may bring financial difficulties faster. This regularity is consistent with the findings arrived at by other researchers, which indicate the correlation between the evaluation of financial situation, being aware of the fact that economic resources are decreasing and the level of fear of COVID-19 [42]. The necessity to take into account the economic context in the rise of the threat posed by the pandemic is indicated by a significant correlation between the fear of unemployment given rise to by the pandemic and the level of fear of COVID-19. This may mean that people experiencing a deep fear of COVID-19 may initially be afraid of losing jobs and income, which as a result may lead to psychological distress.

Subsequent variables measuring the level of deprivation, limitations in life activity, and a decrease in the life status indicate the significance of social consequences of the pandemic. A change in daily habits and lifestyle, imposing quarantine and social distancing, the lockdown of schools, workplaces and national borders have resulted in a social nuisance, at the same time possibly strengthening the sense of fear of the new coronavirus. It is worth adding that a situation of lockdown and the threat to life and health on a mass scale posed by an invisible virus has never occurred in Poland before.

A significant factor influencing mental health during the quarantine is the influence of media, and the estimated probability of becoming infected. People who watch the news about the pandemic more often and experience a higher level of anxiety related to their own health feared COVID-19 more, which is consistent with a lot of results of research [14,39,43,44]. The increased fear of COVID-19 may result from the fact that watching the news depicting death, infected people and the economic crisis may activate fear-related cognitive patterns, which affect the perception of the level of threat posed by the pandemic [7,45].

Psychological variables were a large part of the explanation of the fear of COVID-19 variance. Stress was correlated with fear to the greatest extent, which may mean that fear is a factor causing stress and may lead to depression and mental disorders, which is indicated by numerous research papers [14,33,46]. It is likely that the social effects of the pandemic increase the experience of stress, which consequently increases the level of the experienced fear of COVID-19. It is worth stressing that neuroticism turned out to be statistically insignificant, which may indicate a higher importance of social factors in the rise of mental pathologies during the pandemic. This confirms other findings, which indicate that neuroticism was not correlated with the fear of COVID-19 [47]. It has been shown that problem-focused strategies for coping with stress have impacted on the level of fear of COVID-19. In such cases, it may be assumed that stress has a positive function and activates activities aiming at decreasing the threat. These findings are confirmed in other studies which show the relationship between active strategies and a decrease in psychological distress [48,49]. In this case, fear has an adaptive function and pushes individuals towards taking defensive actions such as wearing protective masks, complying with rules and being motivated to look after one’s health [18,50].

Pessimism has a tendency to increase the levels of fear as well. Pessimistic people will observe more threats and the pandemic will be perceived by them as a more terrible threat than by optimists.

## 5. Conclusions

The first wave of the COVID-19 pandemic had a negative impact on the mental health of the inhabitants of Poland. Demographic, social and psychological occurrences were risk factors leading to a deterioration in mental health. The studies have shown that the secondary effects of the pandemic connected with lockdown were significant in the rise of the fear of COVID-19. Deterioration of social functioning, real and possible loss of resources as well as the exposure to pandemic-related news make society more prone to mental health disorders. It should be expected that the subsequent waves of the pandemic may increase mental disorders in society, and lead to a serious social crisis.

## Figures and Tables

**Table 1:** Characteristics of the research sample.

Variable	Characteristic	*n*	%
Gender	Male	525	49
	Female	554	51
Age	15–24	211	20
	25–34	202	19
	35–44	171	16
	45–54	185	17
	55–64	189	18
	65≥	121	11
Education	Below secondary	180	17
	Secondary	475	44
	Higher	424	39
Evaluation of financial status	Bad	154	14
	Average	343	32
	Good	582	54
Marital status	Single	305	28
	Married	525	49
	Cohabiting	199	18
	Widowed	50	5

**Table 2:** Division of average item values (FCV-19S).

	M	SD
(1) I am most afraid of Corona	2.25 (2.18–2.32)	1.183
(2) It makes me uncomfortable to think about Corona	2.73 (2.66–2.81)	1.230
(3) I worry a lot about coronavirus-19.	2.66 (2.59–2.73)	1.192
(4) Coronavirus-19 is almost always terminal	2.00 (1.93–2.06)	1.027
(5) Coronavirus-19 is an unpredictable disease	3.25 (3.18–3.32)	1.189
(6) My hands become clammy when I think about coronavirus-19	1.89 (1.83–1.95)	1.005
(7) I am afraid of losing my life because of coronavirus-19	2.37 (2.30–2.44)	1.156
(8) When watching news and stories about coronavirus-19 on social media, I become nervous or anxious.	2.47 (2.40–2.54)	1.141
(9) I cannot sleep because I’m worrying about getting Corona.	1.83 (1.77–1.89)	0.988
(10) My heart races or palpitates when I think about getting Corona	2.33 (2.26–2.40)	1.163

**Table 3:** Correlation coefficients.

Variables	2	3	4	5	6	7	8	9	10	11	12	13	14	15	16	17	18	19	20
1. Gender	−0.03	0.03	0.04	−0.09 **	0.02	−0.01	−0.06	−0.12 **	−0.12 **	−0.11 **	−0.01	−0.02	0.05	−0.12 **	−0.14 **	0.02	−0.05	−0.01	−0.03
2. Age		0.30 **	0.00	0.22 **	−0.12 **	−0.12 **	−0.17 **	0.15 **	0.20 **	0.09 **	0.12 **	0.20 **	0.03	−0.28	0.19 **	−0.13 **	−0.23 **	0.10 **	0.14 **
3. Education			−0.06	0.17 **	−0.02	0.17 **	−0.13 **	0.20 **	0.15 **	0.09 **	−0.04	0.11 **	0.04	−0.13	0.09 **	−0.04	−0.11 **	0.10 **	−0.03
4. Evaluation of financial status				0.05	−0.17 **	0.13 **	0.19 **	0.02	0.18 **	0.22 **	0.20 **	0.02	0.07 *	0.28 **	0.00	0.12 **	0.25 **	−0.35 **	−0.17 **
5. Marital status					0.06 *	0.03	0.03	0.05	0.09 **	0.06 *	0.06 *	0.06 *	0.05	−0.06 *	0.03	0.07 *	−0.01	0.05	0.09**
6. Social capital index						−0.01	−0.06	0.00	−0.09 **	−0.16 **	−0.11	0.00	0.03	−0.11	−0.01	−0.07 *	−0.12 **	0.23 **	−0.06 *
7. Loss of economic resources index							0.03	0.18 **	0.20 **	0.19 **	0.09 **	0.09 **	0.15 **	0.09 *	0.14 **	0.15 **	0.14 **	−0.03	0.09 **
8. Fear of losing job								−0.06	0.02	0.11 **	0.04	0.06	0.08	0.21 **	0.06	0.12 **	0.19 **	−0.07	0.22 **
9. Life changes index									0.31 **	0.29 **	0.18 **	0.26 **	0.19 **	0.05	0.43 **	0.06 *	0.09 **	−0.01	
10. Need deprivation index										0.31 **	0.27 **	0.13 **	0.12 **	0.19 **	0.25 **	0.05	0.05	−0.11 **	0.08 **
11. Mindset change index											0.29 **	0.17 **	0.13 **	0.18 **	0.27**	0.12 **	0.16 **	−0.21	0.13 **
12. Life position decrease index												0.08 *	0.03	0.08 *	0.12 **	0.08 **	0.12 **	−0.23 **	0.15 **
13. Level of interest in the information on COVID-19													0.28 **	0.07	0.13 **	0.06 **	0.12 **	0.01	0.42 **
14. Estimated risk of becoming infected with COVID-19														0.19 **	0.16 **	0.11 **	0.29 **	−0.02	0.43 **
15. Neuroticism															0.09 **	0.27 **	0.76 **	−0.42	0.35 **
16. Problem-focused strategy																0.00	0.09 **	0.09 **	0.35 **
17. Emotion-focused strategy																	0.32 **	−0.19 **	0.21 **
18. Stress (K10)																		−0.39	0.46 **
19. Well-being																			−0.08 **
20. (FCV 19S)																			

* *p* < 0.05; ** *p* < 0.01.

**Table 4:** Results of hierarchical regression analysis.

	Model I		Model II		Model III		Model IV	
Variables	β	*p*	β	*p*	β	*p*	β	*p*
Gender	−0.017	0.646	0.014	0.680	−0.035	0.237	0.011	0.686
Age	0.100	0.009	0.171	0.000	0.102	0.003	0.164	0.000
Education	−0.051	0.183	−0.117	0.001	−0.111	0.001	−0.088	0.002
Marital status	0.027	0.467	−0.022	0.526	−0.021	0.503	−0.008	0.556
Evaluation of financial status	−0.144	0.000	−0.061	0.095	−0.088	0.006	−0.051	0.109
Social capital index	0.017	0.651	0.035	0.318	0.018	0.580	0.027	0.326
Loss of economic resources index			0.057	0.125	−0.001	0.986	−0.039	0.186
Fear of losing job			0.298	0.000	0.210	0.000	0.133	0.000
Life changes index			0.225	0.000	0.125	0.000	0.072	0.026
Need deprivation index			−0.101	0.020	−0.113	0.003	0.083	0.017
Mindset change index			0.051	0.233	0.000	0.980	−0.038	0.276
Life position decrease index			0.049	0.180	0.066	0.040	0.071	0.014
Level of interest in COVID-19					0.277	0.000	0.220	0.000
Probability of becoming infected with COVID					0.309	0.000	0.222	0.000
Neuroticism							0.082	0.062
Active strategies							0.144	0.000
Passive strategies							0.033	0.255
Stress							0.318	0.000
Happiness							−0.091	0.007
F (*p* ≤ 0.000)	4.27		14.02		30.97		37.92	
R square	0.026		0.176		0.364		0.489	
Standard error	8.77		8.07		7.09		6.35	

## Data Availability

The data presented in this study are available on request from the corresponding author.
